# Postoperative acromiohumeral interval affects shoulder range of motions following reverse total shoulder arthroplasty

**DOI:** 10.1038/s41598-022-25173-7

**Published:** 2022-12-05

**Authors:** Du-Han Kim, Hyeong-Uk Choi, Byung-Chan Choi, Ji-Hoon Kim, Chul-Hyun Cho

**Affiliations:** Department of Orthopedic Surgery, Keimyung University Dongsan Hospital, Keimyung University School of Medicine, 1035 Dalgubeol-Daero, Dalseo-Gu, Daegu, 42601 Republic of Korea

**Keywords:** Diseases, Health care, Medical research

## Abstract

Reverse total shoulder arthroplasty (RTSA) improves function and reduces pain for patients with complex shoulder problems. However, there is a lack of literature regarding the association of radiographic parameters on clinical outcomes after RTSA. The aim of this study was to analyze various radiographic parameters that may be predictive of clinical outcomes after RTSA. A total of 55 patients treated with RTSA were enrolled. Shoulder radiographic parameters were used for measurement of critical shoulder angle, acromial index, acromiohumeral interval, deltoid lever arm, acromial angulation, glenoid version, and acromial height. Preoperative and postoperative clinical outcomes were evaluated at a minimum 2-year follow-up. An analysis of correlations between radiographic parameters and clinical outcomes was then performed. A significant change in critical shoulder angle, acromiohumeral interval, and deltoid lever arm was observed between preoperative and postoperative radiographic measurements. A significant improvement was observed in all clinical outcomes and range of motions from preoperative to postoperative (all *p* < 0.001). A negative correlation of postoperative acromiohumeral interval with forward flexion (*r* = − 0.270; *p* = 0.046), external rotation (*r* = − 0.421; *p* = 0.001), and internal rotation (*r* = 0.275; *p* = 0.042) was observed at final follow-up. In addition, postoperative acromiohumeral interval less than 29 mm had an 86% positive predictive value of obtaining 130° of forward flexion and 45° of external rotation. It was found that postoperative acromiohumeral interval showed an association with active range of motion in patients who underwent RTSA. In particular, excessive distalization reduced forward flexion and external rotation motion of the shoulder in patients treated with RTSA.

## Introduction

Reverse total shoulder arthroplasty (RTSA), originally designed by Grammont and associates^1^, reduced pain and restored shoulder joint mobility in patients with irreparable massive rotator cuff tears, rotator cuff arthropathy, and other complex shoulder pathologies^2–6^. The indications for RTSA have recently been expanded to include young, higher-demand patients with shoulder problems. Although satisfactory clinical outcomes of RTSA have been demonstrated, development of various complications (e.g., scapular notching, glenoid loosening, acromial stress fracture, and postoperative scapular fracture) can occur, and there are still poor outcomes despite application of consistent technique by the surgeon^7–9^. The original reverse prosthesis design, introduced by Dr. Paul Grammont in 1985, was based on several beliefs: (1) the center of rotation of the glenohumeral joint placed inferior and medial, (2) the prosthesis must be inherently stable, (3) the deltoid lever arm must be effective from the onset of motion^10,11^. Practically, however, there are many difficulties with regard to the methods for ideally assessing implant position in relation to anatomic characteristics.

Patient-related factors and multiple implant-related on both the humeral and glenoid sides play a role in increasing outcomes. Many studies have recently been conducted to improve the design of the RTSA construct and surgical technique to optimize results^12^. Despite various studies, the optimum position of the implant for allowing maximum range of motion (ROM) and outcomes remain debated. For example, Sabesan et al. suggested that deltoid lengthening does not correlate with improvements in active ROM^12^. In contrast, Jobin et al. concluded that deltoid lengthening improves active forward elevation after RTSA^13^.

Furthermore, there are few comprehensive studies of the association between various radiographic parameters and clinical outcomes following RTSA. The aim of this study was to evaluate the relationship between various radiographic parameters and clinical outcomes following RTSA. The hypothesis was that there would be several significant correlations between radiographic parameters and clinical outcomes following RTSA.

## Materials and methods

### Patient selection

**Figure 1:**
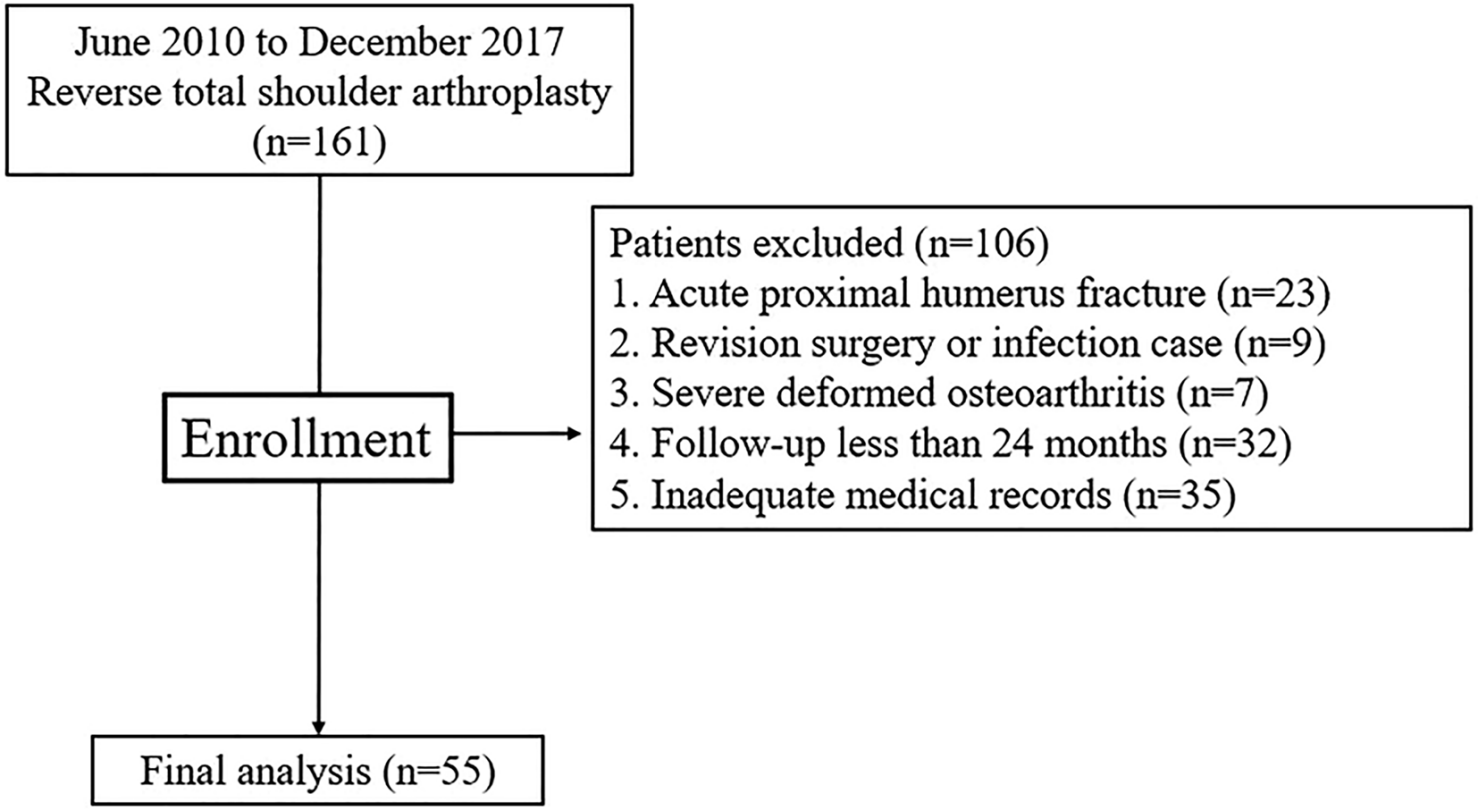
Patient’s flow chart.

This study was approved by the institutional review board of our hospital (IRB No: 2021-04-001). And all methods were carried out in accordance with relevant guidelines and regulartions. A total of 55 patients who underwent RTSA by a single surgeon between June 2010 and December 2017 with a minimum follow-up period of 2 years were included. Inclusion criteria were as follows: (1) patients with cuff tear arthropathy, (2) pseudoparesis with an irreparable massive rotator cuff tear. Painful pseudoparesis was defined as active shoulder forward flexion (FF) < 90° in the presence of full passive forward flexion. Exclusion criteria included patients who: (1) had a fracture or severe deformed osteoarthritis that is difficult to measure, (2) had revision RTSA or infection, (3) inadequate medical records (clinical scores or radiographs) (Fig. 1).

### Surgical procedure and rehabilitation

Surgery was performed with the patients in the beach chair position under general anesthesia using the deltopectoral approach. The TM reverse shoulder system (Zimmer, WaRTSAw, IN, USA) was used in 21 cases, the Aequalis (Tornier, Montbonnot Saint Martin, France) in 21 cases, and the Equinoxe (Exactech, Gainesville, FL, USA) in 13 cases. The shoulder was immobilized in a sling for 6 weeks after surgery. Passive ROM exercises were initiated 2 weeks after surgery. Active ROM exercises were started 6 weeks after surgery.

### Radiographical measurements

Radiographs included a true anteroposterior (AP) view of the glenohumeral joint in neutral rotation and an axial view of a CT scan. Measurements were performed using the Infinitt PACS (Infinitt Healthcare Co., Seoul, South Korea) digital imaging system.

Radiographic parameters including critical shoulder angle (CSA), acromial index (AI), acromiohumeral interval (AHI), deltoid lever arm (DLA), acromial angulation (AA), Glenoid version (GV), and acromial height (AH) were evaluated for each patient before surgery. Postoperative measurements were repeated for CSA, AI, AHI, and DLA.

**Figure 2:**
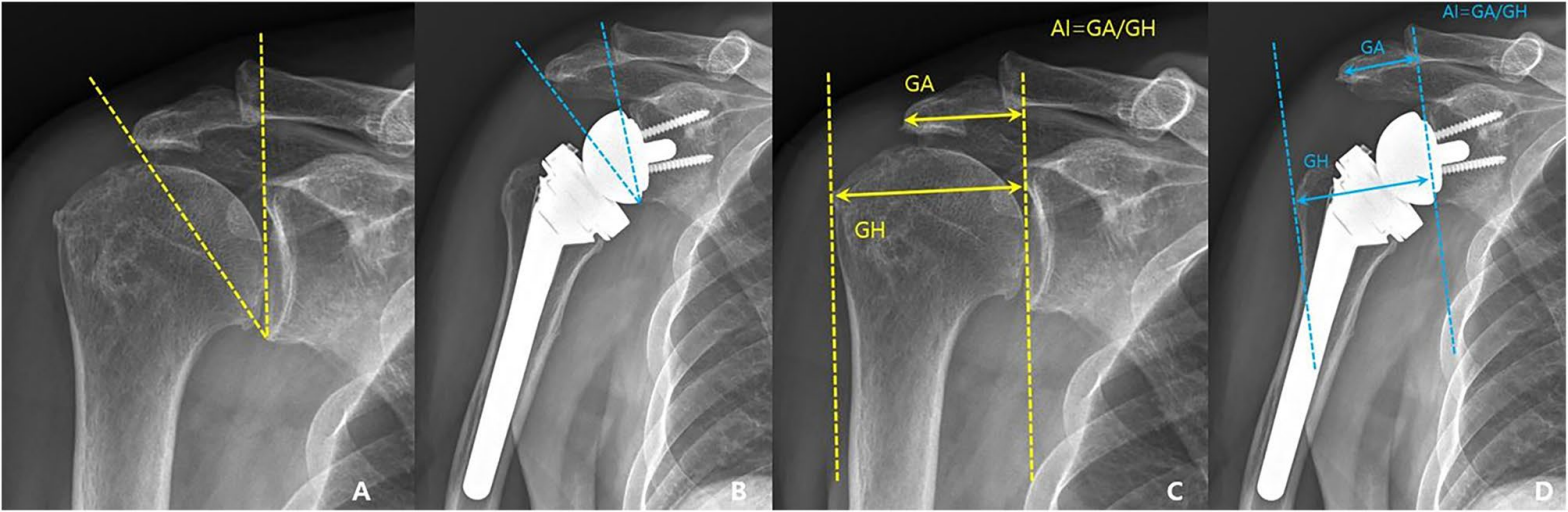
(**A**) preoperative critical shoulder angle, (**B**) postoperative critical shoulder angle, (**C**) preoperative acromial index, (**D**) postoperative acromial index.

**Figure 3:**
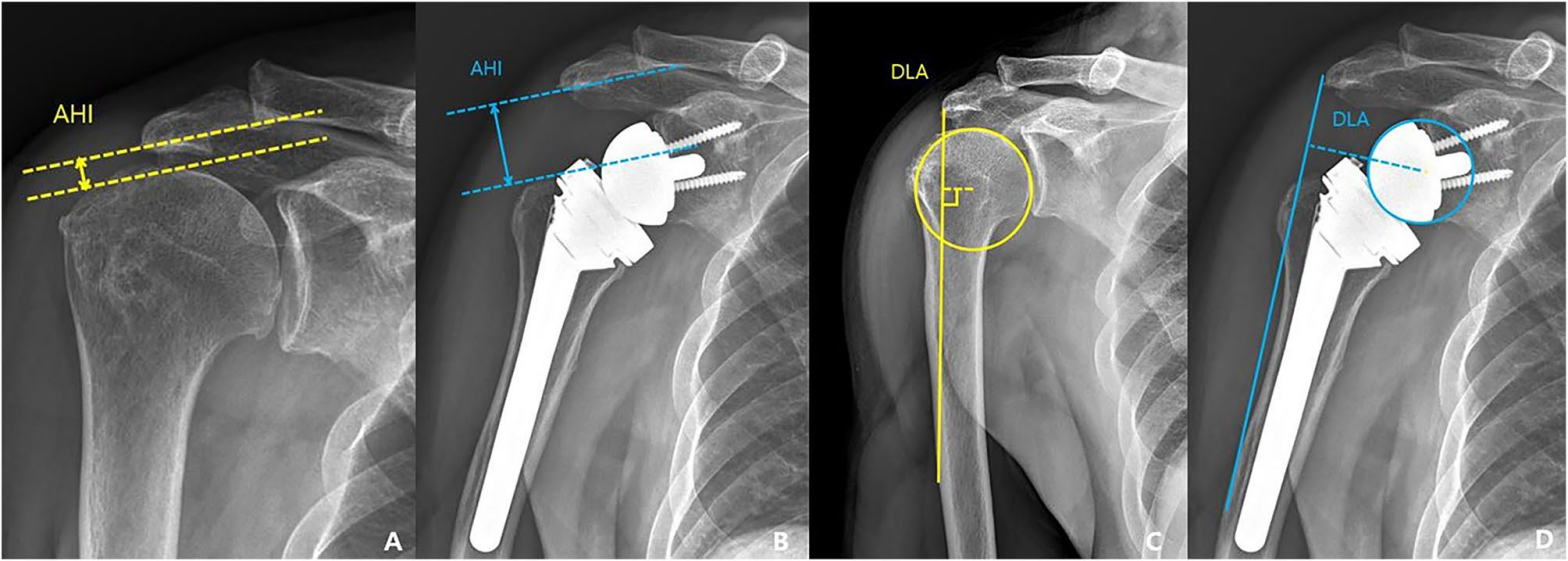
(**A**) preoperative acromiohumeral interval, (**B**) postoperative acromiohumeral interval, (**C**) preoperative deltoid lever arm, (**D**) postoperative deltoid lever arm.

**Figure 4:**
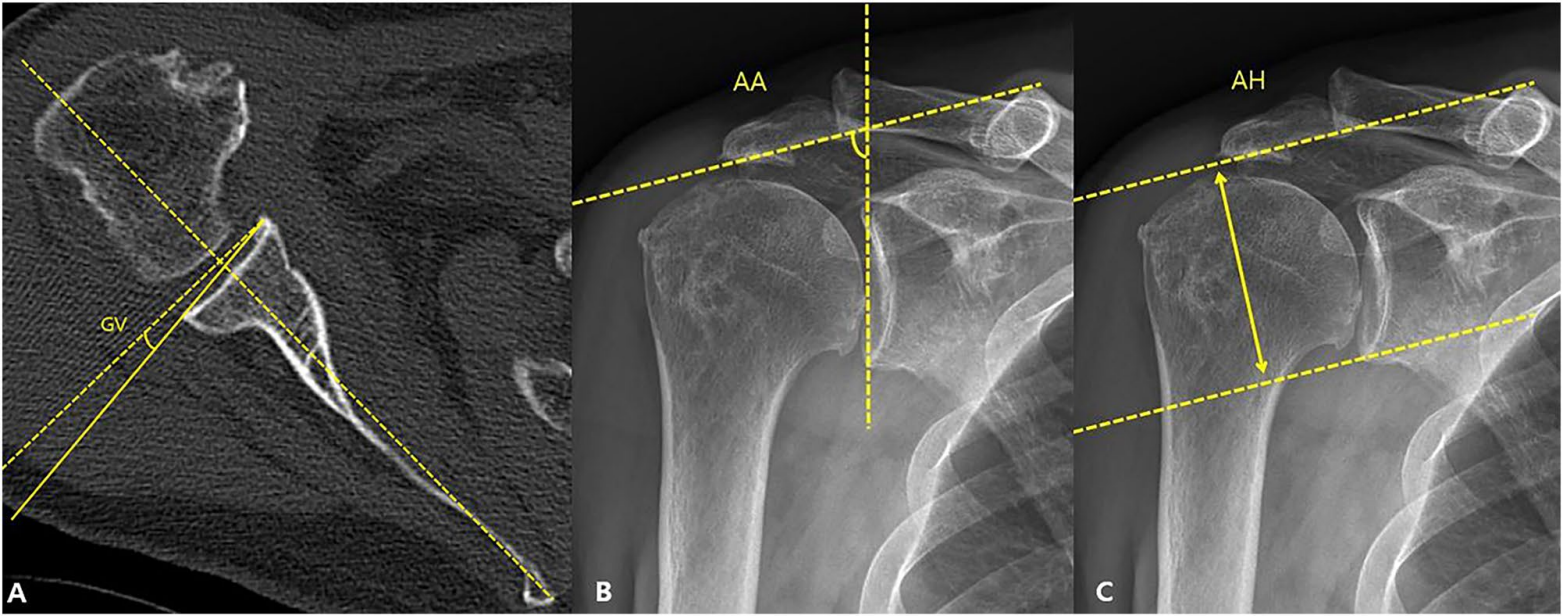
(**A**) glenoid version (**B**) acromial angulation (**C**) acromial height.

The CSA was measured in AP radiographs as described by Moor et al.^14^. The CSA was defined as the angle between a line from the upper to the lower glenoid rim and a second line from the lower glenoid rim to the most lateral acromial extension. The AI was measured as a ratio of the distance from the glenoid to the lateral edge of the acromion over the distance from the glenoid to the lateral edge of the greater tuberosity (Fig. 2)^15^. The AHI was measured by calculating the distance from the undersurface of the acromion to the greater tuberosity perpendicular to the long axis of the acromial body^15^. The DLA was measured from the center of rotation (COR) perpendicularly to a line from the acromion to the deltoid tuberosity^12^. The COR was measured as the center of a best-fit circle of the glenosphere (Fig. 3)^12^. The GV was measured as the angle between the glenoid line and the line perpendicular to the scapular axis^16^. The glenoid line is drawn connecting the anterior and posterior margins of the glenoid fossa, and the scapular axis is drawn from the most medial aspect of the scapula body to the center of the glenoid fossa. The AA was measured in AP radiographs^16^. The AA was defined as the angle between a line from the upper to the lower glenoid rim and a second line set on the undersurface of the acromial roof. The AH was measured in AP radiographs from the most inferior point of the glenoid to the undersurface of the acromial roof (Fig. 4)^17^.

All measurements were performed independently by two orthopedic surgeons blinded to clinical outcomes. And a randomized analysis was repeated by the investigators 4 weeks later for evaluation of interobserver reliability.

### Clinical outcomes and ranges of motion

Clinical outcomes were evaluated using a visual analog scale (VAS) for pain, the University of California at Los Angeles (UCLA) shoulder score, and American Shoulder and Elbow Surgeon (ASES) score. Active ROM was evaluated in terms of FF, external rotation (ER) with the arm at the side, and internal rotation (IR) with the arm at the back. FF was assessed in degrees between the arm and the thorax with the elbow held straight, and ER with 0˚ of shoulder abduction was measured with the elbow in 90˚ of flexion between the thorax and the forearm. IR was measured using an indirect method called “hand behind the back” where the hand was placed behind the back, and the vertebral level reached by the tip of the extended thumb was recorded. For statistical analysis of IR, values were converted into contiguously numbered groups: 1 to 12 for T1 to T12, 13 to 17 for L1 to L5, 18 for the sacrum, and 19 for the buttock^18^. A clinical examination was performed by an independent study coordinator, and these scores and ROMs were obtained at routine preoperative and postoperative clinic visits. We evaluated scapular notching using the Sirveaux classification at final follow-up images and operation related complications^19^.

### Statistical analysis

IBM SPSS ver. 25.0 (IBM Co., Armonk, NY, USA) was used for all data analyses. Inter- and intra-rater reliability were assessed by calculating the Fleiss k correlation coefficient^20^. The interpretation of the strength of agreement determined by the k values was dependent on the criteria of Landis and Koch^21^: values of 0.81 to 1.00 indicate almost perfect agreement; 0.61 to 0.80, substantial agreement; 0.41 to 0.60, moderate agreement; 0.21 to 0.40, fair agreement; and 0 to 0.20, slight agreement. The paired *t*-test was used for comparison of the preoperative and final clinical scores and ROMs. Pearson correlation coefficients were used to examine the relationships between radiographic parameters and clinical outcomes. The variation in ROM and clinical outcomes was examined using receiver operating characteristic curves to establish cut scores for each radiographical parameter that influenced the recorded outcomes. Statistical significance was set at *p* < 0.05 for all statistical comparisons and additionally for the receiver operating characteristic curve analysis of a minimum area under the curve (AUC) > 0.60^15^.

### Ethics declarations

This study was approved by the Institutional Review Board of Keimyung University Dongsan Hospital (IRB No: 2021-04-001).

## Results

Overall, 55 RTSAs were evaluated, including 13 males and 42 females with a mean age of 72.0 ± 5.3 years (range, 60–83 years). The mean follow-up period was 40.0 ± 15.1 months (range, 24–93 months). Prostheses were implanted in 39 right shoulders and 16 left shoulders. Of the patients, 33 were cuff tear arthropathy, and 22 were massive rotator cuff tears (Table 1). Inter-rater and intra-rater reliability were calculated for all measured radiographical parameters. Most of the parameters showed excellent reliability. Good inter- and intra-rater reliability was found for AA, and fair to good reliability was found for GV (Table 2).

**Table 1:** Demographic data.

Clinical characteristic	
Age, year	72.0 ± 5.3
Sex, male/female, n	13/42
BMI, kg/m^2^	24.7 ± 3.8
Follow-up, months	40.0 ± 15.1
Affected arm. right/left, n	39/16
**Diagnosis**	
Cuff tear arthropathy	33
Massive rotator cuff tear	22

*BMI* body mass index.

**Table 2:** Inter- and intra-rater reliability for all radiographic measurements and mean values for radiographic analysis.

	Inter-rater reliability	95% CI	Intra-rater reliability	95% CI	Reliability
Preoperative CSA	0.804	[0.704–0.870]	0.801	[0.699–0.868]	Excellent
Postoperative CSA	0.875	[0.812–0.917]	0.936	[0.901–0.959]	Excellent
Preoperative AI	0.828	[0.740–0.886]	0.893	[0.839–0.929]	Excellent
Postoperative AI	0.894	[0.840–0.930]	0.976	[0.963–0.984]	Excellent
Preoperative AHI	0.734	[0.680–0.772]	0.853	[0.776–0.903]	Good- excellent
Postoperative AHI	0.779	[0.718–0.820]	0.861	[0.788–0.908]	Excellent
Preoperative DLA	0.813	[0.718–0.876]	0.917	[0.875–0.945]	Excellent
Postoperative DLA	0.831	[0.689–0.901]	0.945	[0.901–0.968]	Excellent
Acromial angulation	0.704	[0.618–0.766]	0.719	[0.639–0.776]	Good
Glenoid version	0.451	[0.384–0.499]	0.600	[0.440–0.705]	Fair-good
Acromial height	0.913	[0.868–0.942]	0.935	[0.902–0.957]	Excellent

*CI* confidence interval, *CSA* critical shoulder angle, *AI* acromial index, *AHI* acromiohumeral interval, *DLA* deltoid lever arm.

Overall, a significant change in CSA, AHI, and DLA was observed between preoperative and postoperative measurements. In addition, there was a significant improvement in all clinical outcomes and ROMs from preoperative to postoperative (Tables 3 and 4).

**Table 3:** Radiographic variables: preoperative, postoperative, and change.

	Preoperative	Postoperative	Change	*p-*value
Critical shoulder angle, °	35.9 ± 3.7	31.7 ± 5.3	− 4.2 ± 6.0	0.000*
Acromial index	0.72 ± 0.09	0.70 ± 0.12	− 0.02 ± 0.12	0.310
Acromiohumeral interval, mm	7.3 ± 2.9	27.8 ± 6.0	20.5 ± 5.7	0.000*
Deltoid lever arm, mm	14.2 ± 5.1	41.0 ± 5.1	26.9 ± 5.3	0.000*
Acromial angulation, °	77.1 ± 7.1	–	–	–
Acromial height, mm	54.0 ± 4.5	–	–	–
Glenoid version, °	0.4 ± 3.8	–	–	–

*Statistical significance.

**Table 4:** Clinical outcomes and range of motions: preoperative, postoperative, and change.

	Preoperative	Postoperative	Change	*p-*value
VAS	7.0 ± 2.2	1.2 ± 1.8	− 5.8 ± 2.7	0.000*
UCLA	12.4 ± 4.8	28.4 ± 4.4	16.1 ± 6.2	0.000*
ASES	29.1 ± 15.8	82.4 ± 16.5	53.3 ± 21.1	0.000*
Forward flexion, °	70.0 ± 41.7	141.1 ± 20.5	71.5 ± 44.1	0.000*
External rotation at side, °	22.8 ± 19.6	50.8 ± 13.4	28.0 ± 23.7	0.000*
Internal rotation at back	15.4 ± 2.3	14.2 ± 1.7	− 1.16 ± 2.6	0.000*

*VAS* visual analog scale, *UCLA* University of California at Los Angeles, *ASES* American shoulder and elbow surgeon.

*Statistical significance.

The details of the correlations and associated *p* values are shown in Table 5. Postoperative AHI showed a significant negative correlation with FF (*r* = − 0.270; *p* = 0.046) and ER (*r* = − 0.421; *p* = 0.001), and positive correlation with IR (*r* = 0.275; *p* = 0.042) at final follow-up. In addition, GV showed a significant negative correlation with UCLA score (*r* = − 0.292; *p* = 0.031).

**Table 5:** Correlations and *p*-values of all radiographic measurements and final clinical outcomes.

	VAS score		UCLA score		ASES score		Forward flexion		External rotation		Internal rotation	
	r value	*p-*value	r value	*p-*value	r value	*p-*value	r value	*p-*value	r value	*p-*value	r value	*p-*value
Preoperative CSA	0.010	0.945	− 0.022	0.876	− 0.033	0.809	− 0.036	0.794	− 0.140	0.307	0.185	0.176
Postoperative CSA	− 0.102	0.461	0.108	0.432	0.057	0.681	0.255	0.061	0.008	0.954	0.086	0.531
Acromial index	− 0.028	0.837	0.084	0.543	0.062	0.654	0.038	0.782	− 0.063	0.649	0.141	0.305
Glenoid height	− 0.080	0.560	0.037	0.787	− 0.017	0.901	0.216	0.114	− 0.002	0.990	0.080	0.561
Preoperative AHI	0.009	0.946	− 0.003	0.982	0.029	0.833	− 0.107	0.435	− 0.003	0.985	− 0.002	0.991
Postoperative AHI	0.076	0.582	− 0.176	0.200	− 0.102	0.458	− 0.270	0.046*	− 0.421	0.001*	0.275	0.042*
Preoperative DLA	0.064	0.640	0.029	0.834	− 0.002	0.991	− 0.083	0.545	− 0.055	0.689	0.187	0.172
Postoperative DLA	− 0.010	0.942	− 0.077	0.575	0.009	0.948	− 0.160	0.242	− 0.232	0.089	0.235	0.085
Acromial angulation	− 0.061	0.660	0.063	0.646	0.071	0.608	0.127	0.355	0.092	0.506	− 0.142	0.300
Glenoid version	0.252	0.063	− 0.292	0.031*	− 0.245	0.072	− 0.092	0.504	0.015	0.916	− 0.047	0.731
Acromial height	− 0.070	0.611	0.073	0.599	0.124	0.366	0.047	0.732	0.009	0.950	0.111	0.418

*VAS* visual analog scale, *UCLA* University of California at Los Angeles, *ASES* American shoulder and elbow surgeon, *CSA* critical shoulder angle, *AHI* acromiohumeral interval, *DLA* deltoid lever arm.

*Statistical significance.

**Figure 5:**
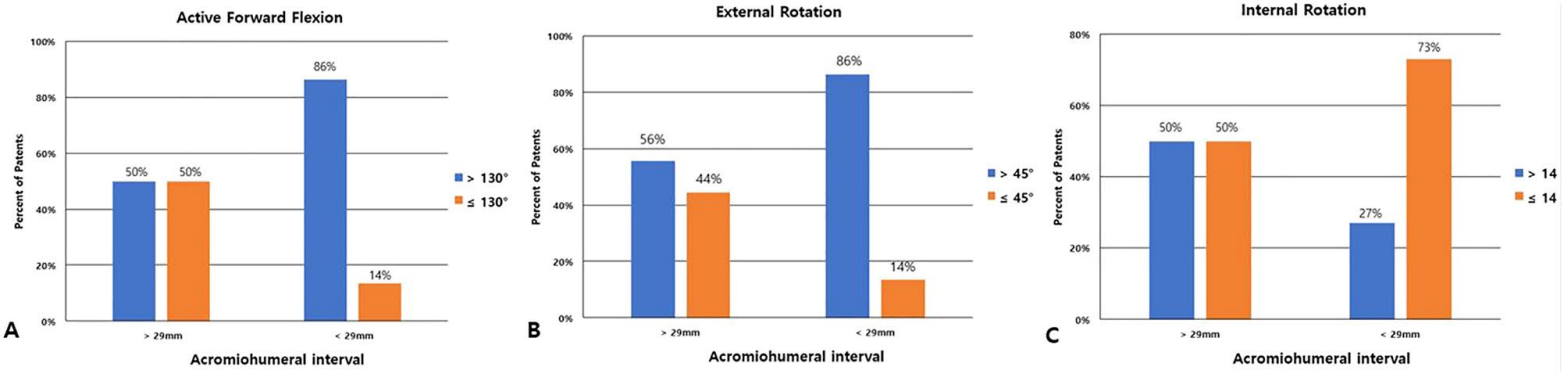
When the postoperative acromiohumeral interval was < 29 mm, then (**A**) nearly 86% of patients achieved > 130° of active forward flexion. (**B**) nearly 86% of patients achieved > 45° of external rotation. (**C**) nearly 73% of patients achieved ≤ 14 of internal rotation.

A contingency table was used to evaluate the predictive value of the postoperative AHI on the ability to obtain 130° of FF, 45° of ER, and 14 of IR. If the AHI was greater than 29 mm, there was a 50% chance of obtaining at least 130° of FF and a 56% chance of obtaining at least 45° of ER. If the distance was less, there was an 86% chance of more than 130° of FF (AUC = 0.688, 95% CI 0.522–0.853, *p* = 0.033) and an 86% chance of obtaining at least 45° of ER (AUC = 0.689, 95% CI 0.515–0.862, *p* = 0.041). However, no significant correlation was found between increased IR and AHI (AUC = 0.612, 95% CI 0.451–0.773, *p* = 0.176) (Fig. 5).

There were no significant difference in clinical outcomes between the 3 prosthesis types. Otherwise, there was a significant difference in ER motion based on implant type. The TM reverse shoulder system and the Equinoxe demonstrated a significant improvement in ER of the shoulder as compared to the Aequalis (*p* = 0.020, *p* = 0.033, respectively). And Aequalis system showed significantly higher values in CSA, AI, AHI, and DLA compared to other implants (*p* = 0.006, *p* = 0.001, *p* = 0.001, *p* = 0.003 respectively).

Scapular notching was observed in 25 shoulders. According to the Sirveaux classification^19^, it was classified as grade 1 in 20 cases, grade 2 in three cases, and grade 3 in two cases. There were three cases of brachial plexus nerve palsy. All patients recovered spontaneously at one, two, and four months after surgery, respectively. However, no severe or systemic complication occurred in any of the patients.

## Discussion

The current study was conducted in order to identify radiographic parameters that are associated with the clinical outcomes in patients after RTSA. The main finding was that postoperative AHI showed an association with ROMs at the final follow-up. However, the other radiographic parameters showed no association with clinical outcomes and ROMs. In particular, we found that 86% of included patients with an AHI of 29 mm or less were able to achieve 130° of FF and 45° ER at the final follow-up.

There is still considerable debate regarding the radiographic parameter of RTSA for optimal restoration of function while maintaining longevity. Distalization of the COR is necessary to provide space for unrestricted ROM of the proximal humerus and tension of the deltoid muscle^22,23^. Deltoid muscle with ideal tension provides the stable fulcrum essential for the active elevation of the shoulder and stability of the prosthesis. However, excessive lengthening is not desirable because it could increase the risk of complications such as neurologic damage, fixed abduction of the arm, or postoperative acromial stress fracture^24–26^. Additionally, failure to restore sufficient deltoid tension may result in poor clinical outcomes^27^. Studies on the appropriate degree of distalization are still in progress and have been conducted using arm length, deltoid length, and AHI.

Ladermann et al. used arm length to confirm correlation with clinical outcomes and retrospectively reviewed 183 RTSA cases^27^. They found no correlation between arm lengthening and improvements in ROM. However, better functional results were achieved with arm lengthening as compared with shortening. In addition, they recommended that arm lengthening above 25 mm compared with the opposite side should not be a surgical objective. Gerber et al. also proposed a 20 to 30 mm lengthening of the arm, as assessed externally with palpation from the tip of the acromion to the elbow^28^.

Deltoid lengthening is recognized empirically as a crucial clinical attribute. Jobin et al. prospectively evaluated 49 patients undergoing RTSA. In their study, a strong correlation was observed between deltoid lengthening and active FF of the shoulder^13^. However, they did not observe over-tensioning related complications or find a plateau effect of deltoid lengthening on FF. A study showing the opposite result has been reported. Sabesan et al. conducted a multi-center study of patients treated with RTSA comparing the relationship between deltoid lengthening with clinical outcomes. As a result, they found that deltoid lengthening showed no correlation with improvements in active FF or ER^12^.

The AHI depends on the thickness and size of the implant, the use of an eccentric glenosphere, and the position of the glenosphere implant in the vertical plane^27^. In a multi-center study involving the AHI, Berthold et al. investigated the prognostic radiographic factors affecting clinical outcomes in patients following RTSA using a 135° prosthesis design^29^. They found that postoperative AHI showed significant positive correlations with FF and the clinical score. Their results were opposed by our study. However, they stated that the exact amount of distalization remains inconclusive.

Although several studies have reported an association of implant design with postoperative clinical outcomes, these results were controversial. Excessive lateralization of the humerus can result in higher forces and soft tissue tension by deltoid muscle, leading to an increased risk of an acromial stress fracture^30–33^. On our radiographic parameters, the AI (glenoid-acromial distance/glenohumeral distance) would represent lateralization of the humerus. Roberson et al. found that postoperative AI of > 0.62 corresponded to ASES score and Penn Shoulder Score, which were 10 points higher than for RTSA patients with an AI of < 0.6^15^. Lateralization of the implant’s COR has been proposed to decrease scapular notching, improve soft tissue tension, and increase impingement-free ROM^33^. DLA or COR of the glenohumeral joint may represent the medializing of the COR. Greiner et al. conducted a prospective study comparing the functional outcomes of non-lateralized versus lateralized RTSA using a 10 mm-autogenous bone graft^34^. They noted a significant improvement in active ER in the lateralized group. However, Jobin et al. found no functional correlation with the COR^13^. In our study, there was a significant difference in radioigraphic lateralization and ER based on implant type. ER could be related to lateralized implant type. The TM reverse shoulder system is a more lateralized glenoid implant than Aequalis. Equinoxe is a more lateralized humeral implant than Aequalis. However, no significant difference in clinical outcomes was observed with the use of these three implant types.

Although postoperative CSA was significantly decreased in our study, there was no significant correlation between CSA and clinical outcomes. Ladermann et al. also reported similar results to our findings. They performed a 3D computer simulation using CT scans of 12 patients with various angles of CSA. Based on 3D computer-based research, they concluded that CSA has no influence on ROM after RTSA^35^.

This study has several limitations. First, it is retrospective in nature and has limitations similar to those of other retrospective studies, and the number of included patients was relatively small (55 patients). Second, we studied only an Asian population of patients who underwent surgery in South Korea. As such, the results may only be generalizable to a similar population. The conduct of further studies in various ethnic groups is needed. Third, we used three different types of implants. Fourth, each patient had a different follow-up period. And we might have measurement bias in this retrospective study even if we tried to select the exact view of radiographs.

Nevertheless, the strength of this study is that the investigation was performed in a homogenous group of patients who underwent RTSA performed by a single surgeon and included comprehensive radiographic parameters. We intend to follow up with this cohort to prove the long-term clinical outcomes and radiographic factors of RTSA.

## Conclusion

Postoperative AHI was found to show an association with active ROM in patients who underwent RTSA. In addition, excessive distalization reduced FF and ER motion of the shoulder in patients who underwent RTSA. Surgeons must be aware and critical of over-lengthening the deltoid tension, which can also have poor outcomes or complications.

## Data Availability

The datasets used and/or analyzed during the current study available from the corresponding author on reasonable request.
